# Ultrasound findings of ovarian intravenous leiomyomatosis: a case report

**DOI:** 10.3389/fonc.2024.1472061

**Published:** 2024-10-28

**Authors:** Jin Li, Le Luo

**Affiliations:** ^1^ Department of Ultrasound, Deyang People’s Hospital, Deyang, Sichuan, China; ^2^ Medical Records Statistics Division, Deyang People’s Hospital, Deyang, Sichuan, China

**Keywords:** intravenous leiomyomatosis, contrast-enhanced ultrasound, ovarian vein, ultrasound examination, case report

## Abstract

Intravenous leiomyomatosis (IVL), an abnormal growth pattern of uterine leiomyomas, is a rare tumor characterized by masses of smooth muscle cells appearing histologically benign and proliferating within the blood vessels but not invading the tissue. Currently, there have been limited reports of early cases of IVL, and the imaging characteristics of IVL remain uncertain, resulting in frequent misdiagnosis prior to surgery. The present study utilized a case of early IVL detected through conventional ultrasound and subsequently confirmed *via* contrast-enhanced ultrasound (CEUS) to further investigate ultrasound’s diagnostic efficacy for early IVL detection. Here, a case of a 49-year-old woman was reported who presented with uterine leiomyoma and an echogenic mass in the left adnexal region on physical examination. Subsequent transvaginal CEUS examination revealed a left ovarian venous leiomyoma. The patient underwent resection of tumors in the uterus, bilateral ovaries, and left ovarian vein under general anesthesia. A venous plexus was identified above the left broad ligament close to the left ovary, and a myoma-like growth was detected in the posterior uterine wall during the operational procedure. Reports on pathology and immunohistochemistry verified leiomyomatosis with fatty metaplasia in the left broad ligament and uterine wall vein. The prognosis of patients with IVL is determined based on the appropriate surgical methods and the timely diagnosis of the condition. In this case, conventional ultrasound aided in the early identification of IVL, which was later verified by a CEUS examination, resulting in a successful surgical treatment. This example highlights the importance of ultrasound technology in diagnosing this uncommon condition and presents a new method for preoperative detection of IVL.

## Introduction

Intravenous leiomyomatosis (IVL) is a rare malignancy characterized by aberrant growth patterns of uterine leiomyomas. Although IVL appears benign during histological examination, it can display aggressive malignant characteristics by spreading beyond the pelvic vein, inferior vena cava, adrenal vein, and renal vein to affect the right ventricular compartment and major pulmonary artery in some instances. This can lead to symptoms such as cardiac murmurs, syncope, and even sudden death (1). Frequent misdiagnosis or delayed diagnosis before surgery, which can result in incorrect medical treatment, is often observed due to the rarity of this condition. Accurate preoperative diagnosis of IVL necessitates comprehensive imaging examination, with computed tomography (CT) or magnetic resonance imaging (MRI) commonly employed due to multifocal lesions involving various locations, including the iliac vein and heart. However, there remains untapped potential for transvaginal and contrast-enhanced ultrasound (CEUS) in the early detection of this disease, which warrants further exploration. Herein, a case has been presented where conventional ultrasound identified intravenous low echo within the ovary that was subsequently evaluated using CEUS, revealing a leiomyoma. This leiomyoma was ultimately confirmed *via* surgical intervention and histopathological analysis as an early-stage IVL case.

## Case presentation

**Figure 1:**
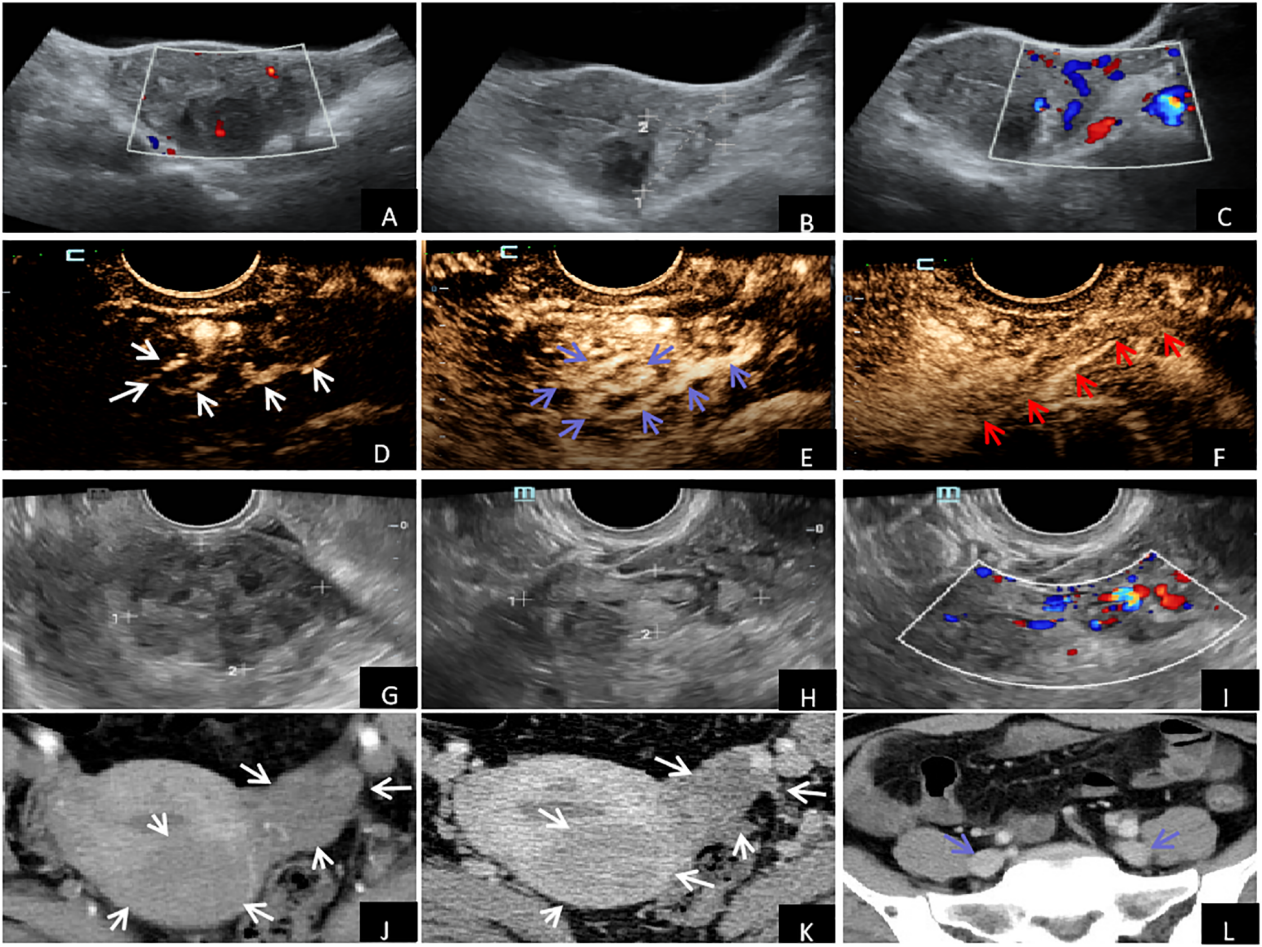
Imaging examination: **(A–C)** Conventional ultrasound examination showed uterine fibroids in the posterior wall, enhanced gyrus mass in the left adnexal area, and tortuous flow signals detected by CDFI. **(D–F)** CEUS showed synchronous and rapid hyperenhancement in the early stage of the arterial mass with enhanced echo in the left adnexal area (white arrow). During the peak stage, the interior enhancement exhibited irregularities resembling the shape of an earthworm (blue arrow). During the venous phase, the lesion exhibited reduced enhancement (hypoenhancement), whereas the outside vessel wall displayed uniform and intensified enhancement (hyperenhancement) (red arrow). **(G–I)** Vaginal ultrasound showed leiomyoma in the posterior wall of the uterus, with cord-like isoecho in the left adnexal area and tortuosity of blood flow signals detected by CDFI. Contrast-enhanced CT of the chest and abdomen of **(J–L)** showed uterine enlargement and leiomyomas in the uterus's posterior and left lateral walls (white arrows). There were no anomalies observed in the iliac vein (blue arrow).

Over 10 months ago, the patient, a 49-year-old female, was developed an irregular menstrual cycle without an apparent cause, which lasted for about 1-2 months, with a menstrual period of 7 days, accompanied by increased menstrual volume, blood clots, no abdominal pain and distension, no frequent urination, and no diarrhea and constipation. Over 5 months ago, the patient received a regular ultrasound examination at our hospital as part of a physical examination (**Figures 1A–C**), revealing leiomyoma of the uterus’s posterior wall with a size of about 40.2 mm × 45.0 mm, with a clear boundary, and a regular shape. An enhanced echo was observed in the left adnexal region, measuring approximately 51.0 mm × 29.5 mmm, with an unclear boundary, particularly concerning the left lateral wall of the uterus. Color Doppler flow imaging (CDFI) detected a meandering flow signal. Pulse wave (PW) measurement and venous flow spectrum were also observed. These findings suggest the presence of a left adnexal ovarian venous leiomyoma. After this, a CEUS examination was recommended for the patient. CEUS examination (**Figures 1D–F**) showed a leiomyoma in the posterior wall of the uterus, and a solid space-occupying lesion in the left ovarian vein course region considered a left ovarian vein leiomyoma. The patient underwent a transvaginal ultrasound examination (**Figures 1G–I**) in our hospital more than 20 days ago. The examination revealed an isoechoic mass located on the posterior wall of the uterus. The mass measures approximately 45.5 mm × 39.9 mm × 44.2 mm in size and showed a clear boundary and regular shape. The CDFI revealed blood flow signals, indicating the presence of a leiomyoma on the posterior wall of the uterus. An elongated, isoechoic structure was identified in the left adnexal region, measuring 53.0 mm × 14.0 mm. The convoluted blood flow signal was observed using CDFI, and the spectrum of venous blood flow was analyzed using PW, indicating the presence of a left ovarian venous leiomyoma. The echocardiography of the heart and ultrasonography of the major arteries in the abdomen revealed no anomalies. The Contrast-enhanced CT scan of the chest and abdomen (**Figures 1J–L**) revealed an enlarged uterus with a 46.2 mm × 43.7 mm isodense mass in the posterior wall. The mass exhibited a relatively low but uniform enhancement, consistent with a diagnosis of uterine leiomyoma. An isodense mass of about 39.6 mm × 29.4 mm was found in the left lateral wall of the uterus, which was slightly enhanced, and the boundary with the left adnexa was not clear. No abnormalities were seen in the iliac vein, inferior vena cava, renal vein, or heart. Summarizing the above analysis, ovarian venous leiomyomas and uterine fibroids were the final diagnoses of the patient.

**Figure 2:**
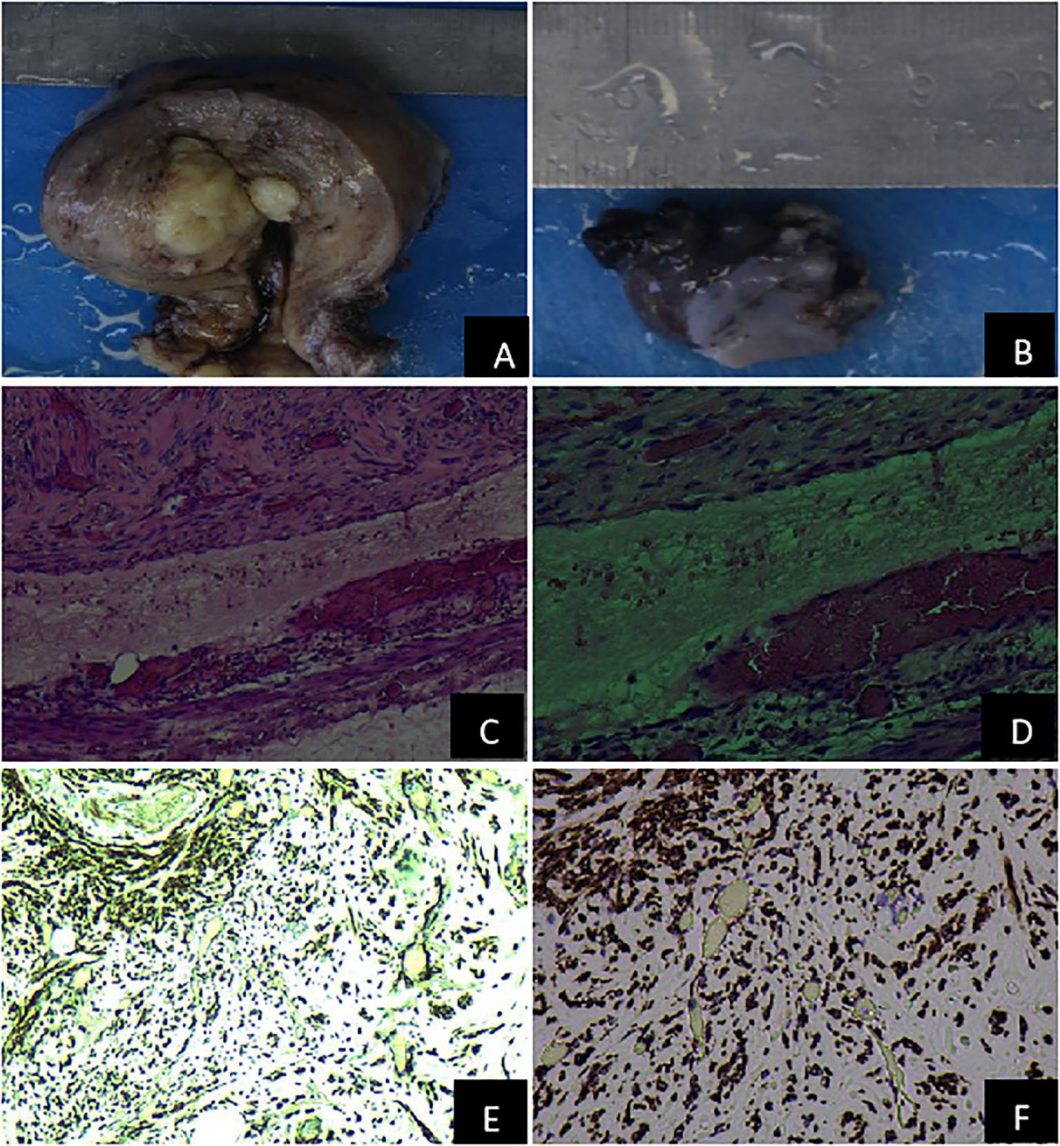
Pathological features of the uterus and the left adnexal mass. **(A)** Gross specimen of the uterus; **(B)** Gross specimen of the left adnexa; **(C)** The pathological examination of venous leiomyoma (H&E 100×); **(D)** The pathological assessment of venous leiomyoma (H&E 200×); **(E)** The immunohistochemical detection of intravenous leiomyoma (Desmin 100×); **(F)** The Immunohistochemical identification of intravenous leiomyomas (Desmin 200×).

The patient underwent resection of tumors in the uterus, bilateral ovaries, and left ovarian vein under general anesthesia. The patient was positioned in lithotomy, and following adequate anesthetic, standard disinfection and draping were performed. An artificial pneumoperitoneum was then established, and laparoscopic surgery was conducted: A leiomyoma-like mass (50.0 mm × 40.0 mm) was observed in the posterior wall, some of which was convex to the serosa layer. A venous plexus (20.0 mm × 30.0 mm) was seen above the left broad ligament near the left ovary, among which a venous tumor was identified. The postoperative cesarean section revealed that the cervix was hypertrophic and had a uniform texture. A fibroid mass of approximately 40.0 mm in diameter was located between the posterior wall of the uterus, exhibiting a whorl-like appearance on the cut surface. The tumor’s sliced surface had a purple-blue coloration. The gross pathological examination (**Figures 2A, B**) revealed that the size of the uterine body was 94.0 mm × 92.0 mm × 61.0 mm. Gray-white nodules measuring 44.0 mm × 32.0 mm × 42.0 mm appeared between the muscle walls. The cut surface was gray-white, solid, and tough, with purple-blue bleeding spots between the muscle walls. The left broad ligament included two nonplastic tissues, measuring approximately 31.0 mm × 21.0 mm × 9.0 mm and 19.0 mm × 14.0 mm × 8.0 mm, which were gray-red and gray-brown in color. The cut surface of these tissues seemed soft and gray-brown. Pathological analysis verified the presence of a spindle cell tumor (**Figures 2C, D**).

Immunohistochemical identification of tumors in the uterus and left broad ligament: Spindle cells were CK (–), CR (–), D2-40 (–), Desmin (+), Caldesmon (+), CD34 (vascular +), CD31 (vascular +), and Ki-67 (+,<5%). When combined with the morphology and immunophenotype of hematoxylin and eosin staining, IVL with fatty metaplasia was observed in the uterine wall and the left broad ligament (**Figures 2E, F**). Throughout the 6-month follow-up, no abnormalities were found in the iliac vessels, great abdominal vessels, or heart.

## Discussion

IVL is a unique and rare tumor that appears histologically benign but has malignant biological behavior. Ordulu et al (2) mentioned that IVL is an atypical intermediary stage between benign and malignant uterine smooth muscle tumors; however, no cases of malignancy have been documented. The cause of IVL is still unclear. Research has indicated a correlation between IVL and elevated levels of estrogen in patients. This condition predominantly affects women who have not yet reached menopause, particularly those with a reproductive history and uterine fibroids (3). There are two views on the mechanism of histological origin of IVL (4, 5). One suggests that it arises from the smooth muscle tissue of the vascular wall. In contrast, the other indicates that it develops from the uterine leiomyoma or myometrium and can aggressively infiltrate nearby venous vessels and spread to distant areas. The patients have been confirmed to have myometrium intravenous vascular leiomyoma and left ovarian intravenous leiomyoma by pathological and immunohistochemical analysis. This suggests that the IVL originated from the uterine fibroids, supporting the second perspective. Some studies have confirmed that phosphoproteome is involved in the inhibition of leiomyoma cell apoptosis and cell proliferation, emphasizing the role of phosphoproteome in leiomyoma growth, which may open up new ideas for the drug treatment of uterine leiomyoma and venous leiomyoma (6).

Early detection and correct diagnosis of IVL are essential for the choice of surgical methods and the prognosis of patients. However, IVL itself has no specific clinical manifestations and is easily ignored because it is often complicated due to the presence of uterine fibroids (3). Early IVL extension occurs within the muscular layer of small blood vessels. It is challenging to detect through imaging examinations. However, in some cases, the tumor may spread to the inferior vena cava and right atrium, or it may be observed during pelvic surgery when a worm-shaped tumor involving the broad ligament is found (7). Correct preoperative diagnosis depends on a large amount of information, especially comprehensive imaging examination. Some scholars tend to recommend advanced chest and abdominal CT, gynecological ultrasound, and MRI as better monitoring methods according to different clinical conditions (8), but it is difficult to differentiate ovarian malignant tumors by imaging diagnosis alone. It is especially difficult to differentiate from uterine sarcoma, which usually lacks specific imaging features and is diagnosed as a presumed benign leiomyoma after hysterectomy or myomectomy. In fact, distinguishing between these diagnoses requires surgical exploration and pathological analysis of resected specimens, which undoubtedly increases the difficulty of leiomyoma treatment and standardized management (9).

IVL was found in the left ovarian vein in the present case. Doppler flow imaging revealed tortuous venous blood flow signals and conventional ultrasound detected an enhanced echogenic mass in the left adnexal area that was not separated from the left lateral wall of the uterus. A transvaginal ultrasound revealed a long tube with isoecho in the left adnexal region, some of which appeared tortuous into clusters. Doppler flow imaging demonstrated tortuous blood flow signals and evaluated the spectrum of venous blood flow. The ultrasound findings mentioned above were in accordance with the information documented in the literature (5). Hence, it is crucial to carefully observe this phenomenon while utilizing traditional ultrasound for physical or screening examinations. This will help distinguish it from ovarian malignant tumors and enhance the early detection rate of IVL. While conventional ultrasonography effectively identifies the site of pelvic veins, it typically struggles to detect minor lesions. Additionally, the limited sensitivity of color Doppler ultrasound prevents the display of lesions in blood perfusion during diagnosis. CEUS provides good visualization of tiny blood vessels and tissue perfusion, compensating for conventional ultrasound’s limitations. IVL CEUS has distinct characteristic manifestations. During the arterial phase, the lesions exhibited fast enhancement, marked by worm-like hyperenhancement. The edge of the lesions displayed a linear and heterogeneous hypoenhancement, with the flowing movement of microbubbles from the edge into the interior. During the venous phase, the lesions decreased in size gradually, exhibiting heterogeneous hypoenhancement. The peripheral wall of the blood vessels displayed homogeneous hyperenhancement, and a marginally thin linear hypoenhancement was observed parallel to the hyperenhancement of the vessel wall. These features are consistent with literature reports (10). Hence, it is crucial to carefully observe this phenomenon during ultrasonographic examination to distinguish it from ovarian malignant tumors and enhance the rate of early IVL diagnosis. In addition, pelvic varicose vein is a gynecological condition that ultrasound can detect. It appears as elongated tubular structures with a worm-like appearance and hypoechogenicity. Blood flow can be observed, except in cases where there is thrombosis. CEUS without the use of contrast agents and IVL can be employed to confirm the presence of thrombosis. Ultrasonography, when compared to enhanced CT and MRI (11, 12), may be helpful for the preoperative diagnosis of IVL. Still, they require longer duration and are associated with the risk of contrast agent allergy, thus limiting their early detection value. Ultrasonic imaging technology is a reliable and safe way of noninvasive radiography and has become the preferred option for screening and diagnosing gynecological diseases. Therefore, improving the understanding of IVL ultrasound imaging is crucial for detecting IVL lesions at an early stage (12, 13). Using CEUS, along with its various features, such as enhancement time, mode, level, and morphology, can significantly improve the comprehension and diagnostic accuracy of IVL (10, 14). This has crucial clinical implications for early intervention and prognosis of IVL.

Ma et al (15) suggested employing echocardiography and CT scanning to evaluate the advancement of intravascular tumors in IVL, provide insight into the preoperative progress of IVL, and aid in developing treatment strategies. Stage I: Disease limited to the pelvic region; Stage II: Lesions present in the iliac vein or inferior vena cava; Stage III: The tumor has invaded the right atrium or ventricle; Stage IV: Cancer has spread to the pulmonary artery. The primary intervention for IVL is surgery; however, there has been ongoing debate on the optimal surgical approach. Depending on the patient’s health, there are two options for surgery: one-stage surgery and two-stage surgery. In severe instances, multidisciplinary collaboration is necessary (15, 16). Given that IVL is a hormone-dependent tumor because of its positive expression of estrogen and progesterone receptors, total hysterectomy (TH) with bilateral oophorectomy (TH-BSO) were previously recommended as the primary surgical approach (17). Moreover, some scholars have shown that compared with transabdominal tumor resection, laparoscopic tumor resection has a variety of benefits, including reducing blood loss, shortening hospital stays, reducing postoperative analgesia needs, and the incidence of complications is not significantly increased (18). Therefore, laparoscopic surgery can be considered as the best treatment for early IVL. However, it depends on the early detection of IVL and making the correct diagnosis. During a regular physical examination, a mixed echoic mass was noticed in the left adnexa of the patient, in the present case study. Based on this finding, it was determined that the mass was an ovarian venous leiomyoma using CEUS. Following a 6-month observation, there was no notable alteration in the lesion. Subsequently, the patient had TH-BSO, and it was determined that the lesion was an ovarian venous leiomyoma. This case shows that transvaginal ultrasound and CEUS technology have great potential in the early detection of this disease, which is consistent with some other researchers who believe that CEUS has higher diagnostic accuracy (14). This significant finding offers novel imaging concepts for the preoperative diagnosis of IVL and warrants further investigation. Simultaneously, sonographers and gynecologists must enhance their understanding of the clinical significance of IVL and ultrasound technology. They should remain attentive in their clinical practice to improve the rate of early diagnosis for IVL.

This case resolved the deadlock on the challenging nature of making a definitive preoperative diagnosis of early IVL based on clinical symptoms and imaging analysis. It introduced a novel imaging approach for diagnosing early IVL. Nevertheless, this case has few limitations since it is subject to the inherent constraints of case reporting. Initially, a comprehensive analysis of imaging pictures of this uncommon ailment, clinical manifestations, and pathological characteristics was not conducted systematically. Furthermore, the patient was monitored briefly, and the outcome in the long run remained uncertain.

## Conclusions

IVL is an uncommon and distinctive tumor, histologically benign but exhibiting malignant biological behavior. Early identification and precise assessment of IVL are essential for choosing suitable surgical methods and evaluating patient prognosis. In this case, conventional ultrasound facilitated early detection of IVL, subsequently confirmed through CEUS examination, leading to a successful surgical intervention. This case highlights the diagnostic significance of ultrasound technology in this uncommon condition and presents a new method for preoperative diagnosis of IVL.

## Data Availability

The original contributions presented in the study are included in the article/supplementary material. Further inquiries can be directed to the corresponding author.
